# Phantom Vision: A Benign, Intraoperative Visual Experience in Cataract Surgery

**DOI:** 10.7759/cureus.57895

**Published:** 2024-04-09

**Authors:** Tejasvi Paturu, John S Jarstad

**Affiliations:** 1 Department of Ophthalmology, University of South Florida Morsani College of Medicine, Tampa, USA

**Keywords:** preoperative anxiety, intraoperative support, intraoperative care, cataract patients, visual experiences in cataract surgery

## Abstract

The intraoperative experience of cataract surgery can be a source of fear and anxiety for many patients. We present a testimonial and illustrations of the intraoperative “phantom vision” experience of a 72-year-old commercial artist during her uncomplicated microincision cataract surgery. She describes a pleasant, colorful, dynamic intraoperative visual experience. First-hand reports from patients can be used in preoperative counseling to reduce anxiety associated with common “visual” phenomena experienced during cataract surgery.

## Introduction

Globally, after refractive error, the second leading cause of vision impairment is cataract [1]. Cataract surgery is the most common procedure in ophthalmic clinical practice: more than half of all Americans over 80 experience cataracts or cataract surgery [2]. Approximately one-third of patients report feeling fear and increased emotional tension prior to their first eye cataract surgery [3]. One study found that over one in four patients experienced intraoperative fear as a result of unanticipated visual sensations during cataract surgery [4]. However, preoperative counseling likely does not involve discussion of intraoperative visual sensations because the literature shows many ophthalmologists do not have knowledge of it and the fear it causes in patients: 54% of United Kingdom ophthalmic surgeons believe that there is no intraoperative visual experience [5].

This case report characterizes the phantom vision that a 72-year-old commercial artist experienced during an uncomplicated microincision cataract surgery. Her written testimonial and painting of her visual experience during cataract surgery can be used in preoperative counseling to alleviate patient anxiety. A broader aim of this report is to characterize the intraoperative visual experience of cataract surgery to the term "transient phantom vision."

## Case presentation

**Figure 1:**
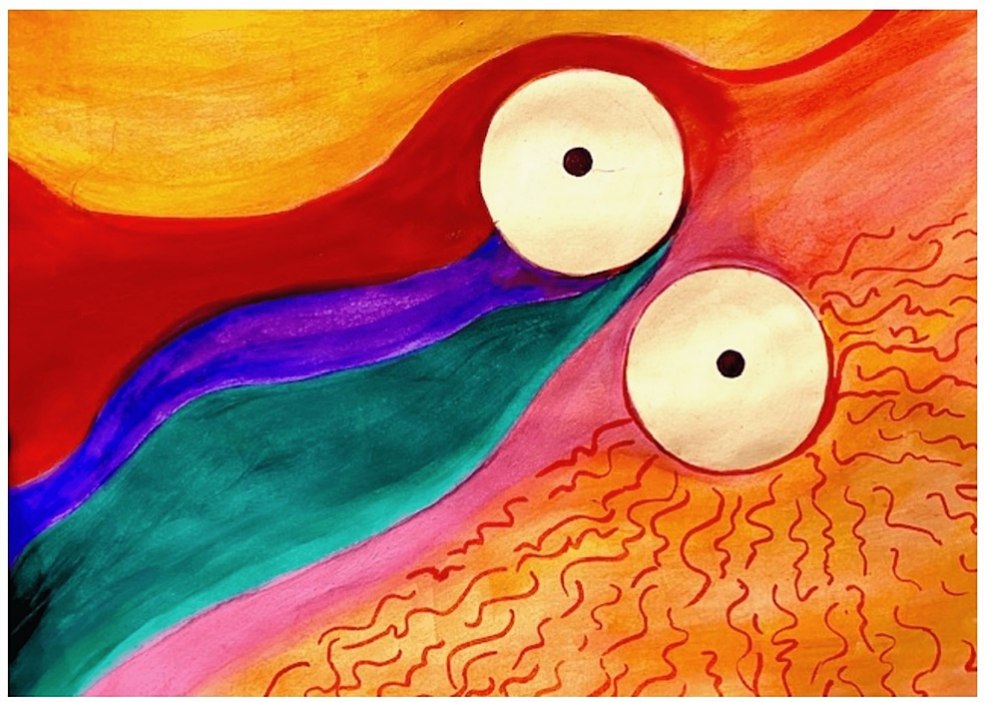
Patient’s painting of intraoperative visual experience during right eye surgery.

**Figure 2:**
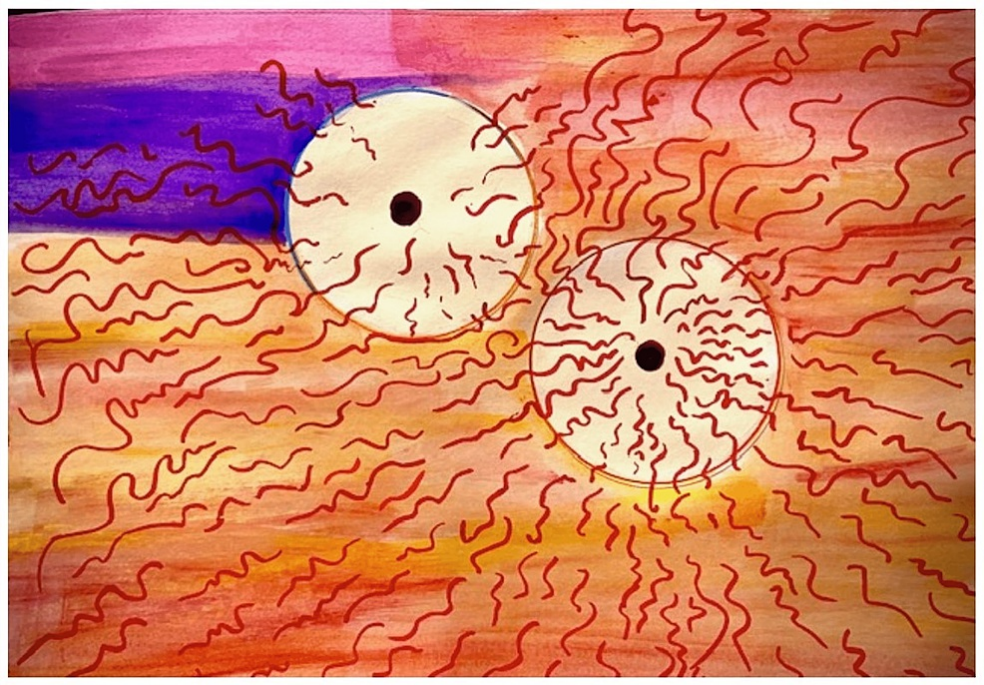
Patient’s painting of intraoperative visual experience during left eye surgery.

The patient is a 72-year-old commercial artist who underwent uncomplicated microincision cataract surgery via standard 2.75 mm clear cornea limbal incision from a temporal approach under standard topical anesthesia in her left and then the right eye. During the procedure, she experienced “transient phantom vision” phenomena in both eyes. She described vivid colors, including bright pink, blue, chartreuse green, yellow, purple, and orange, which swirled and poured into each other during the phacoemulsification portion of her procedure. Her comment during surgery was a single sentence: “Oh, the colors!” At this point, the surgeon asked her to name the various colors she was seeing and any additional shapes. She noticed three bright rectangular objects which we believe represent the three filament lights of the Zeiss microscope light. The colors subsided and disappeared at the conclusion of the procedure and this phenomenon repeated during her second surgery approximately three months later, though slightly different (see Figure 1 and Figure 2). 

In the patient’s own words, “First surgery: Left eye. Dr. Jarstad was emphatic about “keep looking at the overhead lights.” There were three. Each bright white. As I watched they became a brilliant yellow (which) surrounded (each white light). Then the other neon colors started appearing at which point I said out loud that the colors were beautiful and brilliant. They kept swirling and dancing. After a bit, a creamy amoeba shape started moving over the top of the colors. Obscuring and moving to allow peaks of the colors. Then stopping altogether. We discussed this after surgery. It was a singularly amazing experience. I wondered if it could possibly happen with my surgery on my right eye. Second surgery: right eye. Watching the overhead lights I saw the yellow tone around the lights appear again. Then came the colors but more pastel. When the creamy amoeba shape started coming over the colors I asked Dr. Jarstad if he was putting in my new lens he said yes.” 

As described in the patient’s own words, she found the “transient phantom vision” to be pleasant and beautiful, especially upon the surgeon’s reassurance.

## Discussion

Transient phantom vision, similar to phantom limb pain or sensations such as when one’s arm “falls asleep” and it feels like someone is poking needles into the fingers, is reported uncommonly in the literature [6]. In a conversation with David Cogan, MD (National Institute of Health, January 1984), Dr. Cogan described an artist who had surgical removal of one eye for malignant melanoma of the choroid and the artist painted a similar painting of what she “saw” from the side where her eye that had been removed after she closed and covered her remaining sighted eye (David Cogan, oral communication, January 1984). 

Since then, many studies in many different countries have characterized the colorful, intraoperative experience of cataract surgery in groups of patients [4,7-13]. As in our report and others, the images were beautiful and not disturbing to patients and when the surgeon familiar with phantom vision reassured the patient, they were relieved and reassured. The etiology of this phenomenon is not well discussed in the literature. Literature characterizes the different colors, shapes, and intensities of transient phantom vision with respect to demographic and operative variables. However, multiple studies noted that the type of anesthesia used potentially affects the intraoperative visual experience [7-13]. Patients who underwent surgery under topical anesthesia saw more colors and increased vibrancy in comparison to patients who underwent surgery under retrobulbar or parabulbar anesthesia. Retrobulbar or parabulbar anesthesia has been shown to compromise optic nerve function, so the optic nerve may play a role in the etiology of this phenomenon [12]. Additionally, we hypothesize that changes in pressure during the phacoemulsification step may contribute to transient phantom vision. Future developments that provide steady intraorbital pressure may modify or eliminate this phenomenon completely. Documentation of a primary report of this benign intraoperative phenomenon will aid physicians in recognizing and counseling cataract surgery patients to reduce anxiety preoperatively and intraoperatively.

## Conclusions

The growing aging population of the world makes cataract surgery an increasingly common procedure. Given the current population of patients undergoing this procedure frequently reporting pre- and intraoperative anxiety, it is important to include preoperative counseling on what a patient can expect during cataract surgery to improve the patient experience. While previously published literature on the intraoperative visual sensations of cataract surgery are robust and provide a clear picture, we aimed to introduce the term "transient phantom vision" to describe this experience concisely. This literature is also often inaccessible and unused by the elderly patient population undergoing cataract surgery. Although first-hand reports and illustrations such as the ones presented in this report are an accessible and more effective way to convey to patients what they will experience during their surgery, it is important to emphasize that this report is an example and everyone's intraoperative visual sensation will be slightly different. 
